# Carotenoids, β-Apocarotenoids, and Retinoids: The Long and the Short of It

**DOI:** 10.3390/nu14071411

**Published:** 2022-03-28

**Authors:** Earl H. Harrison

**Affiliations:** Department of Human Sciences, Program in Human Nutrition, Ohio State University, 1787 Neil Avenue, Columbus, OH 43210, USA; harrison.304@osu.edu

**Keywords:** vitamin A, retinoic acid, retinoic acid receptors, retinoid X receptors, β-apo-13-carotenone, β-carotene dioxygenases

## Abstract

Naturally occurring retinoids (retinol, retinal, retinoic acid, retinyl esters) are a subclass of β-apocarotenoids, defined by the length of the polyene side chain. Provitamin A carotenoids are metabolically converted to retinal (β-apo-15-carotenal) by the enzyme β-carotene-15,15′-dioxygenase (BCO1) that catalyzes the oxidative cleavage of the central C=C double bond. A second enzyme β-carotene-9′-10′-dioxygenase cleaves the 9′,10′ bond to yield β-apo-10′-carotenal and β-ionone. Chemical oxidation of the other double bonds leads to the generation of other β-apocarotenals. Like retinal, some of these β-apocarotenals are metabolically oxidized to the corresponding β-apocarotenoic acids or reduced to the β-apocarotenols, which in turn are esterified to β-apocarotenyl esters. Other metabolic fates such as 5,6-epoxidation also occur as for retinoids. Whether the same enzymes are involved remains to be understood. β-Apocarotenoids occur naturally in plant-derived foods and, therefore, are present in the diet of animals and humans. However, the levels of apocarotenoids are relatively low, compared with those of the parent carotenoids. Moreover, human studies show that there is little intestinal absorption of intact β-apocarotenoids. It is possible that they are generated in vivo under conditions of oxidative stress. The β-apocarotenoids are structural analogs of the naturally occurring retinoids. As such, they may modulate retinoid metabolism and signaling. In deed, those closest in size to the C-20 retinoids—namely, β-apo-14′-carotenoids (C-22) and β-apo-13-carotenone (C-18) bind with high affinity to purified retinoid receptors and function as retinoic acid antagonists in transactivation assays and in retinoic acid induction of target genes. The possible pathophysiologic relevance in human health remains to be determined.

## 1. Introduction

Humans require a dietary source of vitamin A because they, and other vertebrates, cannot synthesize the retinol, retinal, or retinoic acid required for life-sustaining development and function. Indeed, we now know that vitamin A deficiency or dysregulation of retinoid signaling has profound effects on a vast array of disease processes. Many of these are the subjects of other papers in this Special Issue. The two major forms of dietary vitamin A are long-chain fatty acid esters of retinol, which are found in animal products, and provitamin A carotenoids, which are found primarily in fruits and vegetables. The latter, for example, β-carotene, are enzymatically cleaved to retinal in the intestine, which can then be oxidized to retinoic acid or reduced to retinol.

Carotenoids and apocarotenoids are widely distributed in plants, fungi, algae, and microbes, in which they serve a variety of functions [1]. These include photosynthesis, stress responses, photoprotection, and signaling. In animals and humans, intact carotenoids are believed to function as antioxidants [2].

This review will focus specifically on the occurrence, formation, and function of β-apocarotenoids and their relationship to the retinoids. It first considers the formation of β-apocarotenoids from parent carotenoids by both enzymes and nonenzymatic oxidation. It also reviews the occurrence of β-apocarotenoids in foods and considers similarities in the metabolism of β-apocarotenoids and retinoids. It then focuses on what is known about the direct binding of β-apocarotenoids to nuclear retinoid receptors, RARs, and RXR. Finally, it points out areas that are ripe for further investigation of the relationships between retinoids and other β-apocarotenoids.

## 2. Enzymatic and Nonenzymatic Formation of β-Apocarotenoids

Dietary carotenoids are C-40 polyisoprenoid pigments in fruits and vegetables that give them their yellow, orange, and red colors. Of the over 750 carotenoids found in nature, about 50 are found in the human diet. Some of these (notably β-carotene, α-carotene, and β-cryptoxanthin) are referred to as provitamin A carotenoids since they can be converted in the body to retinol (vitamin A). This conversion involves the cleavage of double bonds of the polyisoprenoid to yield shorter molecules collectively called apocarotenoids [3]. Indeed, the initial product of the cleavage of β-carotene is β-apo-15-carotenal (retinal), which is then enzymatically converted to retinoic acid [4].

**Figure 1:**
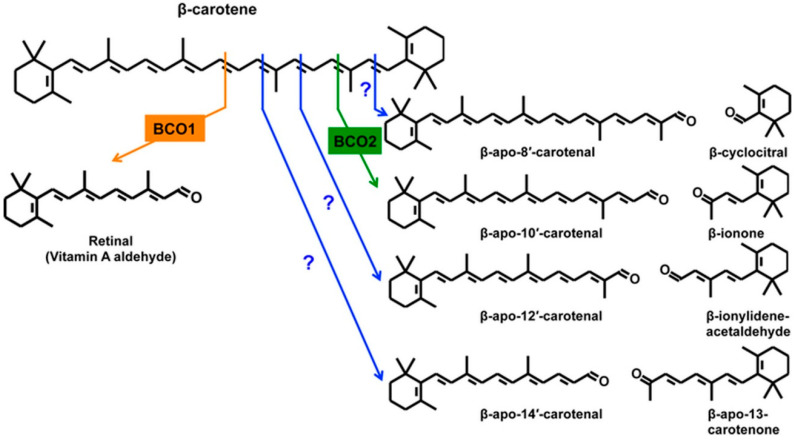
Central and eccentric cleavages of β-carotene. Oxidative cleavage of β-carotene at the 15, 15′ double bond is catalyzed by the enzyme β-carotene 15, 15′-dioxygenase 1 (BCO1) and yields two molecules of retinal (β-apo-15-carotenal). Cleavage at other double bonds leads to the formation of β-apocarotenals and β-apocarotenones. The cleavage at the 9′, 10′ double bond is catalyzed by β-carotene 9′10′-dioxygenase 2 (BCO2) and yields β-apo-10′-carotenal and β-ionone. Eccentric cleavage at other double bonds occurs nonenzymatically or may be enzyme-catalyzed. In cells, the β-apocarotenals can be reduced to the corresponding β-apocarotenols by aldehyde reductases and/or alcohol dehydrogenases. The β-apocarotenals can also be oxidized to the corresponding β-apocarotenoic acids by aldehyde dehydrogenases. (Figure 1 from [4] with permission).

Humans have two enzymes known to catalyze the oxidative cleavage of specific C=C double bonds of dietary carotenoids. β-Carotene-15.15′-dioxygenase (BCO1) cleaves carotenoids having at least one unsubstituted β-ionone ring at the 15-15′ central double bond to yield retinal. β-Carotene-9′,10′-dioxygenase (BCO2) catalyzes primarily the cleavage of the 9,10 or 9′,10′ double bond to yield β-apo-10′-carotenal and β-ionone. Figure 1 shows these conversions using β-carotene as substrate. The figure also shows the products of all other possible C=C double bond cleavages. No other specific vertebrate dioxygenases are known, but it is clear that oxidative cleavage at all positions can occur by chemical oxidation and under conditions of oxidative stress. The relevance of this to human health will be discussed below.

**Figure 2:**
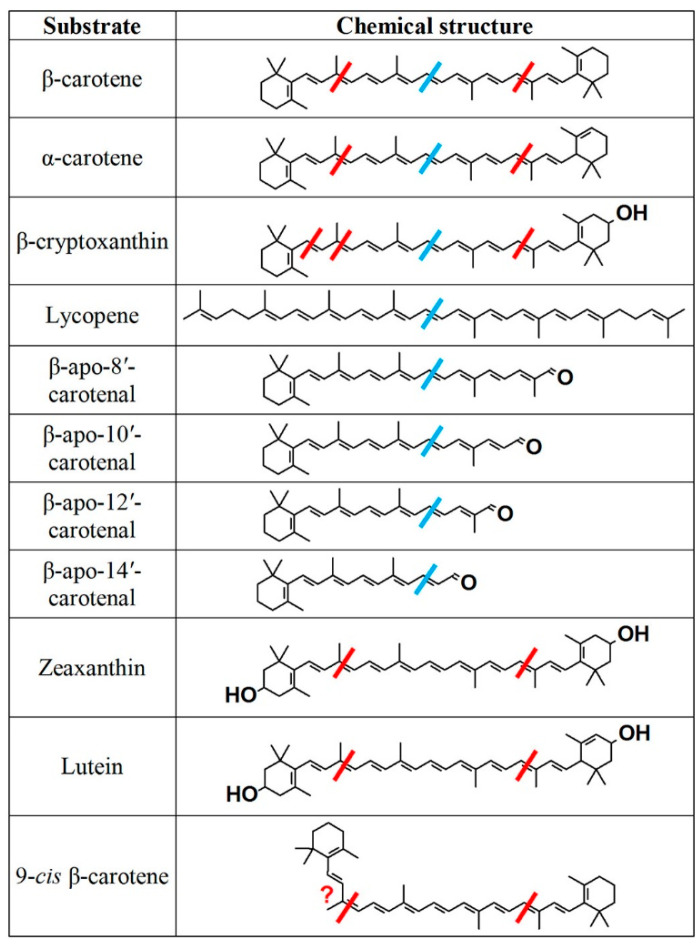
Structures of the substrates tested with purified recombinant chicken BCO2 and human BCO1. The red bars represent the cleavages observed with BCO2, and the blue bars, BCO1. Based on data from references [5,7] and used with permission from [10].

The substrate specificity of BCO1 and BCO2 are compared in Figure 2. BCO1 can utilize any of the substrates tested that have an unsubstituted β-ionone ring, which thus defines those that have provitamin A activity [5]. The enzyme also cleaves all of the β-apocarotenals tested to yield retinal as a product. However, dietary carotenoids with oxygenated terminal rings (viz, the xanthophylls, lutein, and zeaxanthin) are not substrates. Kowatz et al. [6] also expressed human BCO1 and showed it able to catalyze the 15,15′ bond cleavage of β-carotene. They also showed it was a soluble monomeric enzyme in mammalian cells. The substrate specificity shows that BCO1 has an active site that can only accommodate carotenoids with an unsubstituted β-ring (as do α- and β-carotene, β-cryptoxanthin or the β-apocarotenals) or an uncyclized terminus that can adopt such a conformation (as in lycopene).

In contrast to BCO1, BCO2 is a mitochondrial protein that can utilize a larger variety of substrates and cleave them at the 9′,10′ bond. Kelly et al. [8] showed that BCO2 can catalyze the cleavage of both the 9,10 bond and the 9′,10′ bond of β-cryptoxanthin. However, there is much higher catalytic efficiency for cleavage at the 9′,10′ bond. Thus, BCO2 shows strong substrate specificity for substrates with 3-hydroxy-β-ionone rings. This explains why mouse, ferret, and chicken BCO2 catalyze the cleavage of the xanthophylls, lutein, and zeaxanthin [7,9,10]. Research on purified chicken BCO2 agrees with that of Kelly et al. [8] on the substrate specificity of mouse BCO2—namely, the cleavage of the 9,10 and 9′,10′ bond of both provitamin A carotenoids (viz., β-carotene, α-carotene, and β-cryptoxanthin) and of the xanthophylls, zeaxanthin, and lutein [7]. Likewise, the 3-hydroxy substrates are much preferred to β-carotene with its nonhydroxylated rings. A recent study by Bandara et al. [11] included the interesting observation of murine BCO2 catalyzing the cleavage of the 9,10 bond of β-apo-10′-carotenal to yield β-ionone and 10,10′-apocarotene-dialdehyde. Similar results were obtained using apo-10′-carotenoic acid and apo-10′-carotenol. They also presented suggestive evidence that the enzyme cleaved the same bond in the 12′-carotenoic acid and the 14′-carotenoic acid. It remains to be determined whether the BCO1-catalyzed cleavage of the 15, 15′ bond of β-apocarotenals to yield retinoids (retinal) [5] or the BCO2-catalyzed cleavage of the 9,10 bond, which generates products with no vitamin A activity [11] predominates in vivo.

Given the relative activities of BCO1 and BCO2 for provitamin A carotenoids, it appears that BCO1 is the major enzyme involved in the generation of retinal (and thus retinol and retinoic acid) from dietary carotenoids. This contention is also strongly supported by studies using BCO1 null mice. Knockout of BCO1 completely abolished vitamin A production from dietary β-carotene, indicating that BCO1 is both necessary and sufficient for the process [12]. BCO1 null mice also showed small but important effects on whole-body lipid metabolism. The knockout mice developed fatty livers and altered serum lipids, and elevated levels of PPAR-activated genes related to adipogenesis. Thus, the accumulation of tissue carotenoids or their metabolites appears to act as a regulator of lipid metabolism in both liver [12] and adipocytes [13]. Another report demonstrated elevation of hepatic triglyceride accumulation and elevation in PPARγ in BCO1 null mice and demonstrated that BCO1 (both mRNA and protein) was highly enriched in hepatic stellate cells, compared with hepatocytes [14]. BCO1 null mice also have significantly altered lipid and retinoid metabolism in the heart associated with compromised heart function, characterized by reduced contractility [15]. Thus, elimination of BCO1 causes aberrant carotenoid, retinoid, and lipid metabolism and signaling in multiple tissues. Possible mechanisms for the effects of β-carotene metabolites other than retinoids are presented in the final section of this review.

## 3. Occurrence of β-Apocarotenoids in Foods, Blood, and Tissues

β-Apocarotenoids are endogenous components of carotenoid-rich fruits, vegetables, and other plant-derived food as shown by our laboratory [16,17] and others [18,19]. They can also form from autoxidation and thermal degradation of parent carotenoids during food processing or cooking [20,21]. While the levels of β-apocarotenoids vary considerably among different foods in the diet, they always represent small amounts relative to the content of the parent carotenoids. In orange-fleshed melons β-apo-8′, β-apo-10′, β-apo-12′, and β-apo-14′-carotenals and β-apo-13-carotenone were each found at concentrations of 20–40 pmol/gm wet wt. [16], representing about 1–2% of β-carotene. The same β-apocarotenoids (with the addition of β-apo-15-carotenal) were found in a high-β carotene tomato juice, in which they represented only 0.1–0.5% of β-carotene [17].

Some of these eccentric cleavage products have been reported in the serum or tissues of wild-type and BCO knockout mice on control diets or those supplemented with various, and often high, levels of β-carotene or apocarotenoids [14,15,22,23]. The levels in serum and tissues, when detected, are very low, often less than 1% of the parent molecule. Only a small number of studies measuring β-apocarotenoids in human plasma have been reported, most measuring only a few selected compounds. We conducted a randomized, controlled human feeding trial to assess the relationship between dietary and plasma levels of β-carotene and β-apocarotenals in 35 healthy humans [17]. Following consumption of a low-carotenoid diet for two weeks, the subjects then consumed 360 mL daily of a high-β-carotene tomato juice (30.4 mgβ-carotene, 34.5 μg total β-apocarotenals (ones)/day), high-lycopene tomato juice (42.5 mg lycopene, 119.2 μg total lycopenals (ones)/day), or a carotenoid-free cucumber juice for 4 weeks. As expected, plasma levels of β-carotene and lycopene increased in the subjects consuming the corresponding carotenoid-rich juice (retinol levels unchanged). β-Apo-13-carotenone was detected in the blood of all subjects at all visits (0, 2, and 4 weeks). After 4 weeks on the β-carotene tomato juice, it reached a concentration of 1 nM, compared with concentrations of 0.4 nM at baseline and 0.5 nM in controls. Strikingly, β-apo-10′carotenal and β-apo-12′-carotenal were detected in 6 and 2 subjects, respectively, but were below the limit of detection (0.1 nM) in all other subjects. No other β-apocarotenals were detected, suggesting a lack of absorption or very rapid metabolism or degradation.

A recent study followed the formation and absorption of β-apocarotenoids in healthy humans intragastrically and intraduodenally intubated and fed a lipid-rich test meal containing 20 mg of [^13^C-10]-β-carotene [24]. Digesta, plasma, and triglyceride-rich lipoprotein fractions were isolated over 7 h. In the gastric and duodenal contents, small amounts [^13^C]-β-apocarotenals were formed over time, with no detected labeled β-apocarotenols or β-apocarotenoic acids. Expectedly, plasma and TRL fractions contained [^13^C]-β-carotene, [^13^C]-retinyl palmitate and [13]-retinol. No labeled β-apocarotenals/carotenone, β-apocarotenols, or β-apocarotenoic acids were detected. Thus, this study also suggests that there is either no significant intestinal absorption or very rapid degradation of β-apocarotenals in intestinal cells. A final striking result of this study: native (i.e., unlabeled) β-apo-13-carotenone was observed in fasting and postprandial plasma samples from all subjects.

The careful and sensitive analyses of human plasma for β-apocarotenoids just discussed reveal a surprising fact—namely, that only a few long-chain (>20 C) β-apocarotenals, carotenols, and carotenoic acids are detected even when subjects are given a large, single oral dose (20 mg) or even higher daily doses (30 mg) of β-carotene for 4 weeks. In striking contrast, β-apo-13-carotenone (18 C) is found in small quantities (0.5–1 nM) in the plasma of all individuals, even those on low carotenoid diets. We conclude that β-apo-13-carotenone mostly arises from the oxidative cleavage of the 13, 14 C=C bond of the 20 C retinoids, for example, retinol, retinyl esters, retinal and retinoic acid. The unique activity of β-apo-13-carotenone and the β-apo-14′-carotenoids and their possible relevance to retinoid homeostasis are presented in the final section of this review.

The low levels of endogenous β-apocarotenoids, compared with the parent molecules, present both technical and conceptual issues for their investigation. One technical issue is the necessity of highly sensitive and specific methods for analysis, which in practice means using fairly sophisticated LC–MS instrumentation. The other challenge is that many of the β-apocarotenoids and their metabolites are not available commercially in pure form and, therefore, require chemical synthesis. Finally, like the retinoids, many of the compounds are very sensitive to photo- and chemical oxidation. Thus, it is critical to control for the breakdown of the compounds during extraction, sample handling, and during the course of the experiment itself (for example, during incubation in cell culture studies). The latter issue is also a conceptual challenge when using β-apocarotenoids in in vitro experiments. Using the same example, if a cell culture experiment requires 24 h incubation in an oxygen-containing environment, was it the β-apocarotenoid added (and of interest) or an unknown breakdown product that caused the effect? A final conceptual problem is the low endogenous concentrations of most β-apocatotenoids in human and animal tissues. A meaningful mechanistic study requires the use of these physiologically relevant concentrations often in the nanomolar or subnanomolar range.

## 4. Metabolism of β-Apocarotenoids—Similarity to Retinoid Metabolism

Figure 1 presented the structures of all possible products formed from the oxidative cleavage of the double bonds of β-carotene, including the 15,15′ central C=C bond that forms retinal. Cleavage of any of the other bonds thus yields β-apocarotenals (or ketones) with either longer or shorter side chains than retinal. By definition, these are retinoid analogs. Thus, they might be expected to be metabolized by some of the same enzymes or pathways involved in the metabolism of retinal, retinol, and retinoic acid. Indeed, studies conducted so far suggest that this is the case [22].

A number of studies conducted in the 1960s and 1970s focused on the metabolism and in vivo vitamin A activity of β-apocarotenoids, primarily the β-apocarotenals, since at that time, it was unclear whether the relevant pathway of β-carotene conversion to vitamin A (retinal) involved central cleavage of the molecule at the 15, 15′ bond or eccentric cleavage(s) elsewhere. Bagdon et al. [25] administered large daily doses of β-apo-8′-carotenal to dogs and found both the carotenal and β-apo-8′-carotenoic acid in the urine after 14 days. A comprehensive study by Ganguly et al. [26] showed that when β-apo-8′-carotenal was fed to both rats and chickens, blood and tissues contained the carotenal, small amounts of the carotenol, and larger amounts of the carotenoic acid. Olson et al. [27] administered a single oral dose of β-apo-8′-carotenal to humans. Metabolites observed in serum were β-apo-8′-carotenoic acid, β-apo-8′-carotenol, β-apo-8′-carotenyl palmitate, and very minor amounts of the administered β-apo-8′-carotenal. Thus, it appears that both humans and animals readily oxidize dietary β-apo-8′-carotenal to β-apo-8′-carotenoic acid, reduce it to β-apo-8′-carotenol, and esterify the latter to β-apo-8′-carotenyl palmitate.

More recent studies have explored the metabolic fate of β-apo-10′-carotenal, the major product of the BCO2-catalyzed eccentric cleavage of β-carotene. The von Lintig group has clearly shown that WT and BCO1 null mice but not BCO2 null or the double mutant accumulate β-apo-10′-carotenol, mainly in its esterified form, and β-apo-10′-carotenoic acid in the liver when fed β-carotene as the sole source of vitamin A in the diet [22]. They also show that LRAT catalyzes the esterification of apo-10′-carotenol by using RPE microsomes as a source of the enzyme in in vitro assays. The above studies show that some of the β-apocarotenals with longer side chains than retinal can be, such as the latter, oxidized or reduced to the corresponding β-apocarotenoic acids or β-apocarotenols. Whether the same enzymes that carry out these steps in retinoid metabolism catalyze these reactions remains to be determined.

There is also recent evidence for the metabolic formation of the 5,6-epoxides of β-apocarotenols. When Caco2 cells were incubated for 1 h with β-apo-8′carotenal, the compound was largely converted to β-apo-8′-carotenoic acid and to a metabolite identified as 5,6-epoxy-β-apo-8′-carotenol [28]. The cells used do not express BCO1, nor do they esterify retinol unless supplemented with exogenous fatty acids. Identical experiments with β-apo-10′-carotenal led to the production of 5,6-epoxy-apo-10′-carotenol and a minor metabolite that is likely dihydro-β-apo-10′-carotenol. The 5,6-epoxidation of β-apocarotenoids thus mimics this same metabolic fate known for the retinoids. It is not known if the same enzymes are involved.

Given the not-too-surprising similarities in the metabolism of retinal (β-apo-15-carotenal) and the longer chain β-apocarotenals, it is tempting to speculate that the latter could act as competitive inhibitors of the metabolism of the former. Thus, excessive production of β-apocarotenoids (e.g., under oxidative stress) could thereby directly disrupt retinoid homeostasis.

As many of the enzymes that metabolize retinol, retinal, and retinoic acid preferentially utilize the retinoid substrates bound to a specific retinoid-binding protein, determination of the affinities of the β-apocarotenoids to the RBPs is important. There is evidence that β-apo-10′-carotenol bound to RBP4 is taken up and esterified by cells expressing STRA6 and LRAT, albeit at a much-reduced rate than RBP-bound retinol [22].

## 5. β-Apocarotenoids as Ligands for RAR and RXR

There are a large number of in vivo animal studies and in vitro cell studies on the effects of carotenoids and apocarotenoids on growth, differentiation, embryonic development, gene expression, and cell signaling in vertebrates, as detailed in recent reviews [29,30]. These studies suggest a number of regulatory pathways are involved in the effects observed after feeding or dosing with apocarotenoids. Often the results suggest the possible involvement of nuclear receptors and, in some cases, retinoid receptors. In this section, we focus specifically on those studies providing information on the direct effects of β-apocarotenoids on retinoic acid receptors and retinoid X receptors.

During studies on β-carotene and adipocyte differentiation, Ziouzenkova et al. [31,32] presented evidence that β-apo-14′-carotenal acted as a transcriptional repressor of RARα, RXRα, PPARα, PPARγ, LXRα, and LXRβ. They used standard NR-LBD-Gal4 reporter assays in transfected cells. They showed that apo-14′-carotenal (but not β-apo-8′-carotenal) at concentrations of 5–10 μM inhibited agonist-induced activation of these receptors. Quantitatively, the inhibition was greatest for RXRα-LBD activated by LG100364 at 300 nM and PPARα-LBD activated by WY14643 at 10 μM. We investigated the effects of all possible oxidative cleavage products of β-carotene, both as the apocarotenals and apocarotenoic acids, on RXRα [33]. RXRα and RXRE-luciferase constructs were transfected into COS-1 cells and treated with the compounds. None of them activated RXRα. However, β-apo-13-carotenone led to greater than 90% inhibition of reporter activation by 10 μM 9-cis-retinoic acid. Only modest inhibition was observed for the other β-apocarotenoids. We also found that as little as 1 nM β-apo-13-carotenone shifted the dose–response curve for the activation of RXRE-luciferase by 9-cis-retinoic acid. Competitive radioligand binding assays with purified RXRα showed that β-apo-13-carotenone bound to the receptor with the same affinity as 9-cis-retinoic acid (7–8 nM) [34].

**Figure 3:**
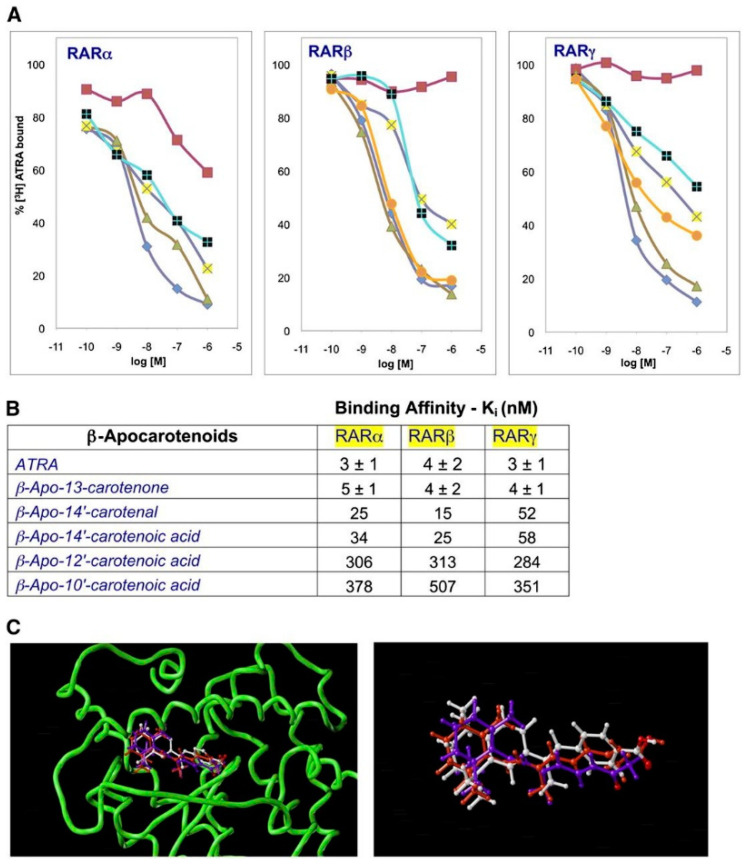
β-Apo-13-carotenone is a high-affinity ligand for purified retinoic acid receptors and fits into the ligand-binding site: (**A**) competitive displacement of 5 nM tritiated atRA from purified RAR proteins by unlabeled atRA (filled diamond) as a positive control, β-apo-13-carotenone (filled triangle), 14′-CA (plus), 14′-AL (cross), and 13-cis-RA (filled square) as a negative control for the RARα (left) experiment, and CD 2665 (filled circle) and retinyl acetate (filled square) as a negative control for RARβ (middle) and RARγ (right) experiments; (**B**) binding affinities (in nM) of β-apocarotenoids to RARs calculated from the data shown in (**A**) and additional experiments with β-apo-12′-and β-apo-10′-carotenoic acids. For atRA and β-apo-13-carotenone, variance shown is for three independent experiments; (**C**) molecular modeling of the docking of atRA (red) and β-apo-13-carotenone (purple) into the ligand-binding site (protein backbone in green) of RARβ (PDB entry:1xap) (left). Shown on the right is the energy minimized then docked conformations of atRA (red) and β-apo-13-carotenone (purple) overlaid onto the conformation of the agonist TTNPB (white) as observed in the X-ray structure. Taken from reference [34] and used with permission.

The effects of β-apocarotenoids on RARs were also studied [34]. Again, none of the β-apocarotenoids significantly activated RARs. However, β-apo-13-carotenone, β-apo-14′-carotenal, and β-apo-14′-carotenoic acid all antagonized the retinoic-acid-induced activation of RARs. All were high-affinity ligands for purified RARα, RARβ, and RARγ (Figure 3A,B). Indeed, β-apo-13-carotenone had an affinity for all three receptors identical to retinoic acid (3–5 nM). Molecular modeling revealed that β-apo-13-carotenone can bind to the ligand-binding site of RARs (Figure 3C). Finally, the three β-apocarotenoids significantly inhibited retinoic acid-induced expression of RARβ and CYP26A1 (two direct targets of retinoic acid) in HEPG2 cells, with the greatest inhibition observed with β-apo-13-carotenone.

## 6. Summary and Future Directions

Retinoids are a subclass of β-apocarotenoids, distinguished chemically by the length of the polyene side chain and the oxidation state of the terminal carbon (hence “the long and the short of it” in the title). Both are formed and metabolized by two carotenoid cleaving dioxygenases found in vertebrates including humans. Polymorphisms of both enzymes in humans are associated with important diseases [4]. A variety of β-apocarotenoids are found in foods, and their levels can increase by nonenzymatic oxidation during storage, food processing, and cooking. Current evidence suggests that there is little absorption of intact β-apocarotenoids in the intestine. Nonetheless, characterization of their interaction with other phytochemicals in the gut and effects on the luminal environment is warranted. It is clear that β-apocarotenals, carotenols, and carotenoic acids are metabolized to products analogous to those that form from the retinoids. An important question to answer is whether the same enzymes are involved. It would be important to determine if β-apocarotenoids bind to retinoid-binding proteins that are critical in chaperoning retinoids during their transport, delivery to cells, and function. While it is clear that C-18 and C-22 β-apocarotenoids bind with high affinity to both RARs and RXR, it is critical to determine the physiological and pathophysiological relevance in humans. It is likely that these β-apocarotenoids will increase in concentration under conditions of severe oxidative stress. Thus, studies of animal models and human diseases characterized by oxidative stress are called for. Given that β-apocarotenoids, like retinoids, are chemically labile and present in small concentrations, it is important to ensure against their generation or degradation during experimentation. Continued development of sensitive and specific methods for their synthesis and analysis will help in assessing all of these areas.

## Data Availability

Not applicable.
